# Supplementary material for: Widespread Reassortment Contributes to Antigenic Shift in Bluetongue Viruses from South Africa
Source: Viruses. 2023 Jul 23;15(7):1611. doi: 10.3390/v15071611 (PMC10383083; doi:10.3390/v15071611)
Supplement: Supplementary file 1 [file viruses-15-01611-s001.zip › viruses-2515212-supplementary.pdf]

Supplementary Table S1. BTV isolates used in this study as well metadata concerning the country of origin, year of submission and GenBank accession numbers.

Virus serotype	Isolate name	Country of origin	Year of isolation	Accession number
BTV-1	BTV-1_VR27_RSA_2017	Ermelo, MP, RSA	2017	MG255599 - MG255608
BTV-1	BTV-1_VR49_RSA_2017	Edenburg, FS, RSA	2017	MG255659 - MG255668
BTV-1	BTV-1_VR13_RSA_2017	Elliot, EC, RSA	2017	MG255549 - MG255558
BTV-1	BTV-1_VR06_RSA_2017	Queenstown, EC, RSA	2017	MG255519 - MG255528
BTV-2	BTV-2_VR18_RSA_2017	Queenstown, EC, RSA	2017	MG255569 - MG255578
BTV-2	BTV-2_VR62_RSA_2017	Beaufort West, WC, RSA	2017	MG255689 - MG255698
BTV-2	BTV-2_VR04_RSA_2017	Bethal, MP, RSA	2017	MT028379 - MT028388
BTV-3	BTV-3_VR05_RSA_2017	Queenstown, EC, RSA	2017	MG255509 - MG255518
BTV-3	BTV-3_VR22_RSA_2016	Bethal, MP, RSA	2016	MT028399 - MT028408
BTV-3	BTV-3_VR11_RSA_2017	Wesselsbron, FS, RSA	2017	MG255539 - MG255548
BTV-3	BTV-3_VR33_RSA_2017	Smithfield, FS, RSA	2017	MG255619 - MG255628
BTV-4	BTV-4_VR01_RSA_2017	Rosendal, FS, RSA	2017	MG255489 - MG255498
BTV-4	BTV-4_S74 _RSA_2016	Ceres, WC, RSA	2016	MG255459 - MG255468
BTV-4	BTV-4_VR24_RSA_2017	Wesselsbron, FS, RSA	2017	MG255579 - MG255588
BTV-4	BTV-4 _VR39_RSA_2017	Smithfield, FS, RSA	2017	MG255639 - MG255648
BTV-4	BTV-4_4wtrus_RSA_2011	Rustenburg, NW, RSA	2011	KT317665 - KT317674
BTV-4	BTV-4_VR30_RSA_2017	Smithfield, FS, RSA	2017	MG255609 - MG255618
BTV-4	BTV-4_VR31_RSA_2017	Bloemfontein, FS, RSA	2017	MT043208 - MT043217
BTV-4	BTV-4_VR03_RSA_2017	Bethal, MP, RSA	2017	MG255499 - MG255508
BTV-4	BTV-4_VR42_RSA_2017	Smithfield, FS, RSA	2017	MG255649 - MG255658
BTV-4	BTV-4_VR34_RSA_2017	Smithfield, FS, RSA	2017	MG255629 - MG255638
BTV-4	BTV-4_VR48_RSA_2017	Vryheid, KZN, RSA	2017	MT043258 - MT043267
BTV-5	BTV-5_Benoni_RSA_2015	Benoni, GP, RSA	2015	MG255449 - MG255458
BTV-5	BTV-5_VR45_RSA_2017	Queenstown, EC, RSA	2017	MT043248 - MT043257
BTV-7	BTV-7_VR43_RSA_2017	Beaufort West, WC, RSA	2017	MT043228 - MT043237
BTV-12	BTV-12_VR44_RSA_2017	Queenstown, EC, RSA	2017	MT043238 - MT043247
BTV-12	BTV-12_VR16_RSA_2017	Elliot, EC, RSA	2017	MT028389 - MT028398
BTV-12	BTV-12_VR54_RSA_2017	Queenstown, EC, RSA	2017	MG255669 - MG255678
BTV-12	BTV-12_VR55_RSA_2017	Queenstown, EC, RSA	2017	MG255679 - MG255688
BTV-13	BTV-13_Prieksa_03_RSA_2014	Prieska, NC, RSA	2014	MG255469 - MG255478
BTV-13	BTV-13_VR38_RSA_2017	Smithfield, FS, RSA	2017	MT043218 - MT043227
BTV-16	BTV-16_VR08_RSA_2017	Bethal, MP, RSA	2017	MG255529 - MG255538
BTV-17	BTV-17_Prieksa_07_RSA_2014	Prieska, NC, RSA	2014	MG255479 - MG255488
BTV-17	BTV-17_VR17_RSA_2017	Queenstown, EC, RSA	2017	MG255559 - MG255568
BTV-24	BTV-24_VR25_RSA_2017	Beaufort West, WC, RSA	2017	MG255589 - MG255598
